# Transcriptional activation of HIF-1 by a ROS-ERK axis underlies the resistance to photodynamic therapy

**DOI:** 10.1371/journal.pone.0177801

**Published:** 2017-05-17

**Authors:** María Julia Lamberti, María Florencia Pansa, Renzo Emanuel Vera, Martín Ernesto Fernández-Zapico, Natalia Belén Rumie Vittar, Viviana Alicia Rivarola

**Affiliations:** 1 Departamento de Biología Molecular, Facultad de Ciencias Exactas Físico-Químicas y Naturales, Universidad Nacional de Río Cuarto, Río Cuarto, Córdoba, Argentina; 2 Schulze Center for Novel Therapeutics, Mayo Clinic, Rochester, Minnesota, United States of America; Duke University, UNITED STATES

## Abstract

Photodynamic therapy (PDT), a promising treatment option for cancer, involves the activation of a photosensitizer (PS) by local irradiation with visible light. Excitation of the PS leads to a series of photochemical reactions and consequently the local generation of harmful reactive oxygen species (ROS) causing limited or none systemic defects. However, the development of resistance to this promising therapy has slowed down its translation into the clinical practice. Thus, there is an increase need in understanding of the molecular mechanism underlying resistance to PDT. Here, we aimed to examine whether a relationship exists between PDT outcome and ROS-involvement in the resistance mechanism in photosensitized cancer cells. In order to recapitulate tumor architecture of the respective original tumor, we developed a multicellular three-dimensional spheroid system comprising a normoxic periphery, surrounding a hypoxic core. Using Me-ALA, a prodrug of the PS PpIX, in human colorectal spheroids we demonstrate that HIF-1 transcriptional activity was strongly up-regulated and mediates PDT resistant phenotype. RNAi knockdown of HIF-1 impairs resistance to PDT. Oxidative stress-mediated activation of ERK1/2 followed PDT was involved on positive modulation of HIF-1 transcriptional activity after photodynamic treatment. ROS scavenging and MEK/ERK pathway inhibition abrogated the PDT-mediated HIF-1 upregulation. Together our data demonstrate that resistance to PDT is in part mediated by the activation of a ROS-ERK1/2-HIF-1 axis, thus, identifying novel therapeutic targets that could be used in combination with PDT.

## Introduction

Photodynamic therapy (PDT) is an emerging antitumoral approach consisting in the administration of a photosensitizer (PS) followed by local irradiation with visible light of specific wavelength(s). In the presence of oxygen molecules, the light illumination of PS can lead to a series of photochemical reactions and consequently the generation of harmful reactive oxygen species (ROS). Thus, interaction of PS, light, and oxygen can selectively induce three main anti-tumor mechanisms: direct tumor cell killing, damage of the tumor vasculature and post-treatment immunological response [1].

Protoporphyrin IX (PpIX), synthesized from 5-aminolevulinic acid (ALA), is an endogenous and safe PS, which accumulates in tumors following ALA or its derivatives (e.g. Me-ALA) administration [1–3]. There have been reports of eradication followed by photodynamic treatment using PpIX and other PS, however some data reveal responses to PDT within the first weeks, but long-term recurrence [4–9]. Multiple hypothesis have been postulated to explain the mechanism of this unfavorable outcome [10–12], however, the molecular events underlying this cellular event remains for the most part elusive.

In this study, we developed a hypoxic avascular colorectal model by the generation of *in vitro* three dimensional (3D) spheroids. We demonstrated a role for the hypoxia inducible factor-1 (HIF-1), master regulator of oxygen homeostasis, in the resistance to PDT. Further analysis of the mechanism showed that HIF-1 is activated through a ROS-ERK1/2 pathway. Finally, we demonstrated that upregulation of HIF-1 in the center of 3D cultures diminished PpIX accumulation, which was at least in part responsible to the PDT resistance.

## Methods and materials

### 2.1. Cell culture

SW480 human colorectal adenocarcinoma were grown in complete medium DMEM (Dulbecco’s modified Eagle medium high glucose 1X, Gibco) supplemented with 10% v/v fetal bovine serum (FBS) (PAA Laboratories), 1% v/v glutamine (GlutaMAXTM 100X Gibco), 1% v/v antibiotic (Penicillin 10,000 units/mL—streptomycin 10,000μg/mL Gibco) and 1% v/v of sodium pyruvate 100 mM (Gibco). Cells were maintained in 5% CO_2_ and 95% air at 37°C in a humidified incubator. Stock cultures were stored in liquid nitrogen and used for experimentation within 5 to 7 passages. SW480 cells constitutively expressing HRE-GFP construct (SW480-HRE) were generated by stable transfection of them with a plasmid containing the gene for destabilized EGFP placed under the control of a promoter region consisting of five copies of a 35-bp fragment from the HRE of the human VEGF gene and a human cytomegalovirus (CMV) minimal promoter. The plasmid was kindly provided by Dr. Foster (Universidad de Rochester, USA) [13]. SW480 cells constitutively expressing GFP (SW480-G) were generated by stable transfection of them with pZsGreen1-N1 (Clontech) plasmid. Transfection was performed using FuGENE^®^ HD Transfection Reagent (Roche) according to manufacturer´s instructions. Stable transfected cells were selected in growth medium supplemented with 2 mg/ml of active Geneticin (G418) (Life Technologies). SW480-HRE cells constitutively expressing siRNA against human HIF-1α mRNA (SW480-HRE shHIF) were generated by lentiviral infection of pLKO.1-shRNA-HIF-1α-1 plasmid. The plasmid was kindly provided by Dr. Eric Metzen (University of Duisburg-Essen, Germany) [14]. Generation of recombinant lentivirus was carried out as described previously [15]. Stable transfected cells were selected in growth medium supplemented with 2 μg/ml of Puromycin (Sigma). Individual colonies were isolated after 2–3 weeks of growth under selection using the cloning ring method and subsequently expanded into clonal cell lines. Expression of GFP was assessed under fluorescence microscope [16]. Hypoxic condition was achieved by incubating cells on a hypoxia chamber (Thermo Fischer Scientific) at 37°C, 5% CO_2_, and 1% O_2_ for 24 h. Chemically induced hypoxia was performed by exposing cells cultured in normoxic conditions (21% O_2_) to CoCl_2_ (Sigma-Aldrich, St. Louis, MO, USA) for 24 h.

### 2.2. Photodynamic treatment

SW480 cells monolayers were washed twice with PBS to remove all traces of FBS and then incubated with Me-ALA (Sigma) in growth medium without FBS for 4. SW480 cells growing as spheroids were taken out from agarose-coated plates, put on plates without agarose and washed twice with PBS before Me-ALA addition during 24 h, in growth medium without FBS.

After Me-ALA incubation, monolayer or spheroids were irradiated at room temperature with monochromatic light source (636 nm ± 17 nm) using a MultiLED system (coherent light). The fluence rate was 0.89 mW/cm^2^, as measured by Radiometer Laser Mate-Q. Drug solution was then removed and replaced with fresh medium [17].

### 2.3. Cell viability assay

Cell viability was evaluated by 1-(4,5-dimethylthiazol-2-yl)-3,5-diphenylformazan (MTT) assay, which is reduced by mitochondrial dehydrogenases of viable cells to non water-soluble violet formazan crystals [18]. Twenty four hours post-PDT, MTT solution (5mg/ml in phosphate buffer saline, PBS) was added for 4 h (dilution rate: 1/10). Then, dimethyl sulfoxide (DMSO) was added to lyse the cells and solubilize the precipitated formazan product. Optical density of the resulting solution of formazan salt was read at 540 nm using ELISA reader plate (Thermo Scientific, Multiskan FC).

### 2.4. Three dimensional cultures

Spheroids were generated using liquid overlay technique. Briefly, an agarose stock solution (1%) was prepared in H_2_O and sterilized by autoclaving. Prior to seeding of the cells, bottoms of 96-well plates were coated with agarose. After cooling for 0.5 h, the plates were ready to use. Cell suspensions were prepared, allowing the seeding of 20000 cells in a total volume of 100 μL[19]. Using this protocol, spheroids were generated with a homogeneous size distribution of similar diameter (923.75 +/- 40.07 μm, n = 13).

### 2.5. Confocal and epifluorescence microscopy

Confocal and epifluorescence imaging experiments were carried out on an inverted Olympus FV1000 (Spectral) confocal microscope (CIQUIBIC-UNC-CONICET) and on an inverted Carl Zeiss fluorescence microscope (UNRC) coupled to a high resolution monochromatic digital camera, respectively. Images were then analyzed using the ImageJ software (1.46r version).

### 2.6. Determination of ROS levels

ROS levels were measured by using the fluorescent dye 2´7´-dichlorodihydrofluoresceindiacetate (H_2_DCFDA) (Sigma), which is a nonpolar compound converted into a non-fluorescent polar derivative (H_2_DCF) by cellular esterases after incorporation into cells. H_2_DCF is membrane-impermeable and is oxidized rapidly to the highly fluorescent 2´,7´-dichlorofluorescein (DCF) in the presence of intracellular ROS [20]. For the experiments, 30 min after treatment, tumor spheroids were incubated in phenol red-free medium and 7 μM HD_2_CFDA dissolved in dimethyl sulfoxide (DMSO) was added. After an incubation of 30 min, intracellular DCF fluorescence (corrected for background fluorescence) was evaluated by fluorescence microscopy. DCF fluorescence was quantified using ImageJ 1.42q software.

### 2.7. Immunoblot analysis

Total cell lysates were extracted with lysis buffer containing 20 mM HEPES pH 7.5; 1.5mM KCl; 1 mM EDTA; 1 mM EGTA; 0.15% Triton-X100; 1 mM PMSF; 1 mM DTT; and a cocktail of protease inhibitors (Sigma). As HIF-1α is very quickly degraded, it required a special protocol for isolation (for example, ice cold conditions) [21]. The protein content of the lysate was measured using BCA protein assay reagent (Pierce). Aliquots containing equal amount of protein were separated by SDS-PAGE and then transferred onto PVDF membranes (Sigma). Blots were blocked with 5% nonfat dry milk in PBS Tween 0.1% (PBST) and then incubated with primary antibodies overnight: anti-HIF-1α antibody (R&D–MAB1536), anti-β-actin (Calbiochem–CP01), anti-phospho-ERK1/2 (Santa Cruz, sc-7383), anti-ERK1/2 (Santa Cruz, sc-292838), anti-phospho-Akt (Cell Signaling, 9271), anti-Akt (Cell Signaling, 9272). Next, blots were incubated with corresponding horseradish peroxidase–conjugated IgG secondary antibody (anti-rabbit or anti-mouse, Cell Signaling). Immunoreactive bands detection was carried out using the enhanced chemoluminescence (ECL) kit (Amersham) according to the manufacturer’s instructions.

### 2.8. Flow cytometry

Reporter expression was determined 12 h after PDT. Spheroids were harvested and disaggregated by trypsinization. Single cells were analyzed on a FACScalibur microflow cytometer (Becton Dickinson). Using forward and side scatter parameters, dead cells and debris were eliminated from the analysis. GFP + cells represented HIF + cells. Next, GFP intensity was measured on HIF + population, indicating HIF transcriptional activity of this cells[22]. Data was analyzed using FlowJo 10.0.7 software (TreestarInc, Ashland, US).

### 2.9. Intracellular PpIX quantification

For the measurement of intracellular PpIX content, the medium was removed, the spheroids were washed twice with PBS, disaggregated by tripsinization and washed with PBS again. After that, cells were brought into extraction solution consisting of a mixture of methanol-1 N percloric acid (1:1, v/v). After 15 min of incubation the resulting solution was centrifugated (1000 xg) and fluorescence intensity was measured in supernatant by means of the spectrofluorimeter (Fluoromax-3 JobinYvon-Horiva, λ_exc_: 405 nm, λ _em_: 601 nm). PpIX fluorescence was normalized to cellular concentration [23].

### 2.10. Statistical analysis

Differences between groups were tested by 1 or 2-way analysis of variance with Bonferroni post-hoc tests using Infostat software. All the results are expressed as mean +/- standard error of at least three independent experiments, and P<0.05 was considered statistically significant. References of figures: P<0.05 = *; P<0.01 = **; P<0.001 = ***.

## Results

### 3.1. 3D tumor architecture lowers the efficacy of photodynamic treatment

**Fig 1:**
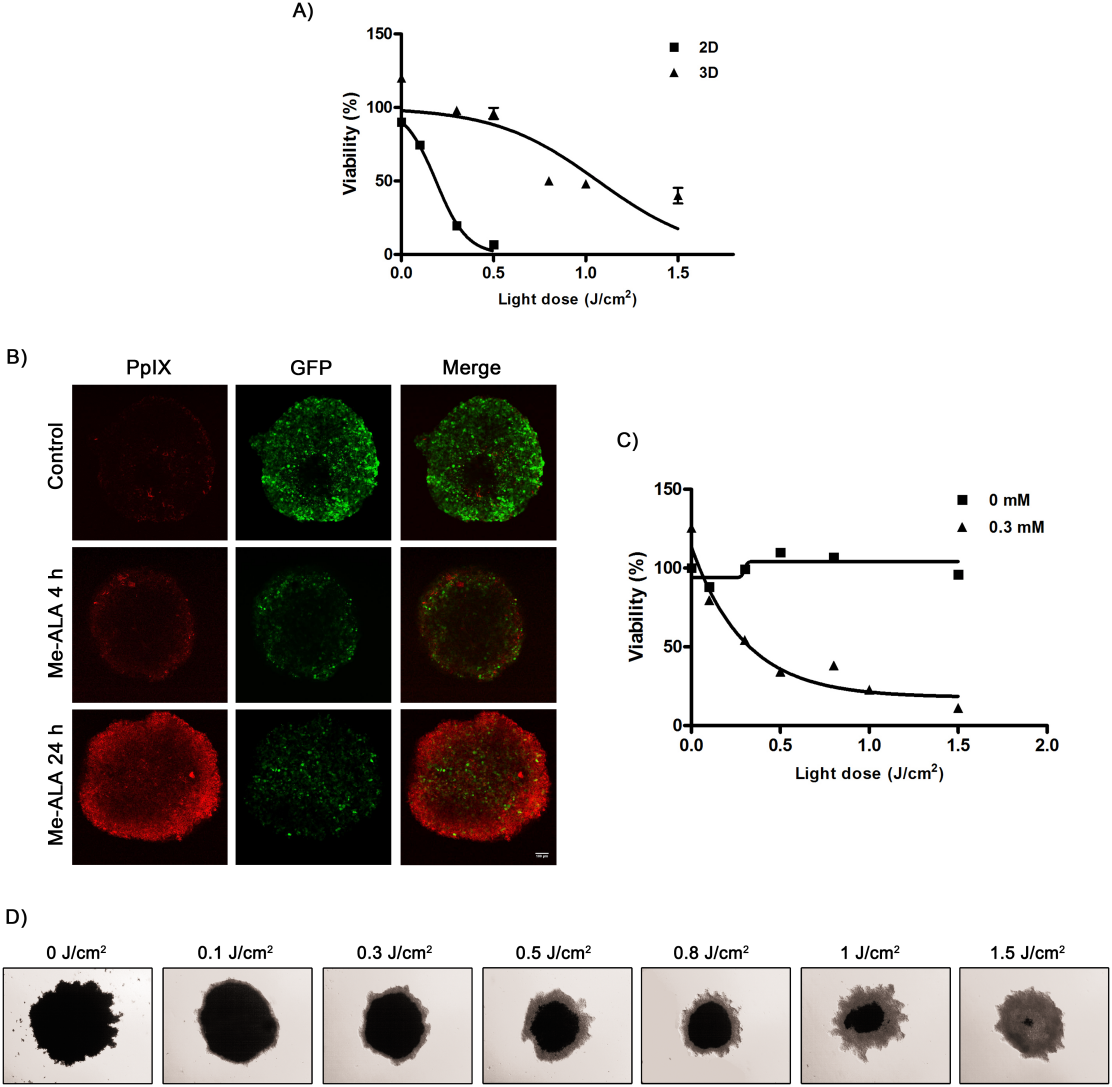
Three-dimensional spatial configuration of colorectal tumor cells confers resistance to photodynamic therapy. (A) SW480 cells growing as monolayers (2D) or spheroids (3D) were incubated with Me-ALA (0.3 mM) for 4 h and then exposed to irradiation (0–1.5 J/cm^2^). Viability was evaluated by MTT assay 24 h post-PDT and referred to non-treated (no light, no Me-ALA) conditions (data not shown). (B) Representative confocal microscopy images of the central layer of SW480-G spheroids incubated with Me-ALA (0.3 mM) for 4 and 24 h. SW480-G tumor cells (green) constitutively expressed GFP; PpIX shows red fluorescence. Bar = 100 μm. (C) SW480 spheroids were incubated with Me-ALA (0.3 mM) for 24 h and then exposed to irradiation (0–1.5 J/cm^2^). Viability was evaluated by MTT assay 24 h post-PDT and referred to non-treated (0 mM Me-ALA, 0 J/cm^2^) conditions. (D) Representative light microscopy images of MTT-incubated, untreated and PDT-treated spheroids 24 h after PDT. Viable areas (MTT +) are dark as formazan salt. References: 2D: Two-dimension cultures (monolayers); 3D: Three-dimension cultures (spheroids).

As tissue oxygenation status is a parameter that defines PDT efficacy [1], 3D cultures constitute an ideal system for investigating photodynamic outcome. To mimic the tumor O_2_, pH, and nutrients gradients we used as model for our photodynamic therapeutic protocol the SW480 spheroids, mimicking hypoxic areas of colorectal cancer (~900 μm from the oxygen source). Spheroids were sensitized with Me-ALA-induced PpIX and subsequently irradiated. 3D cultures treated using the same photodynamic conditions than their 2D counterparts show less than 50% of cell death (Fig 1A). To effectively kill tumor spheroid, drug administration protocol was modified. Pre-treatment experiments were carried out to determine the optimal incubation time with Me-ALA. 3D cultures were generated from SW480-G, which constitutively express GFP to help identify viable cells (GFP+) [24]. From these studies, it was observed that the ideal experimental design included the application of the prodrug during 24 h before irradiation. In contrast to the previously applied treatment regimen (4 h Me-ALA incubation), there was a significant conversion of Me-ALA to PpIX at 24 h incubation, even though its distribution was not homogeneous (Fig 1B). Importantly, this prolonged application of Me-ALA followed by irradiation was able to decrease SW480 spheroids viability dose-dependently (Fig 1C). Those layers found near the center of the spheroid (MTT positive (MTT+), dark) were unaffected by photodynamic activity (Fig 1D).

### 3.2. PDT-induced oxidative stress promotes the activation of ERK signaling

**Fig 2:**
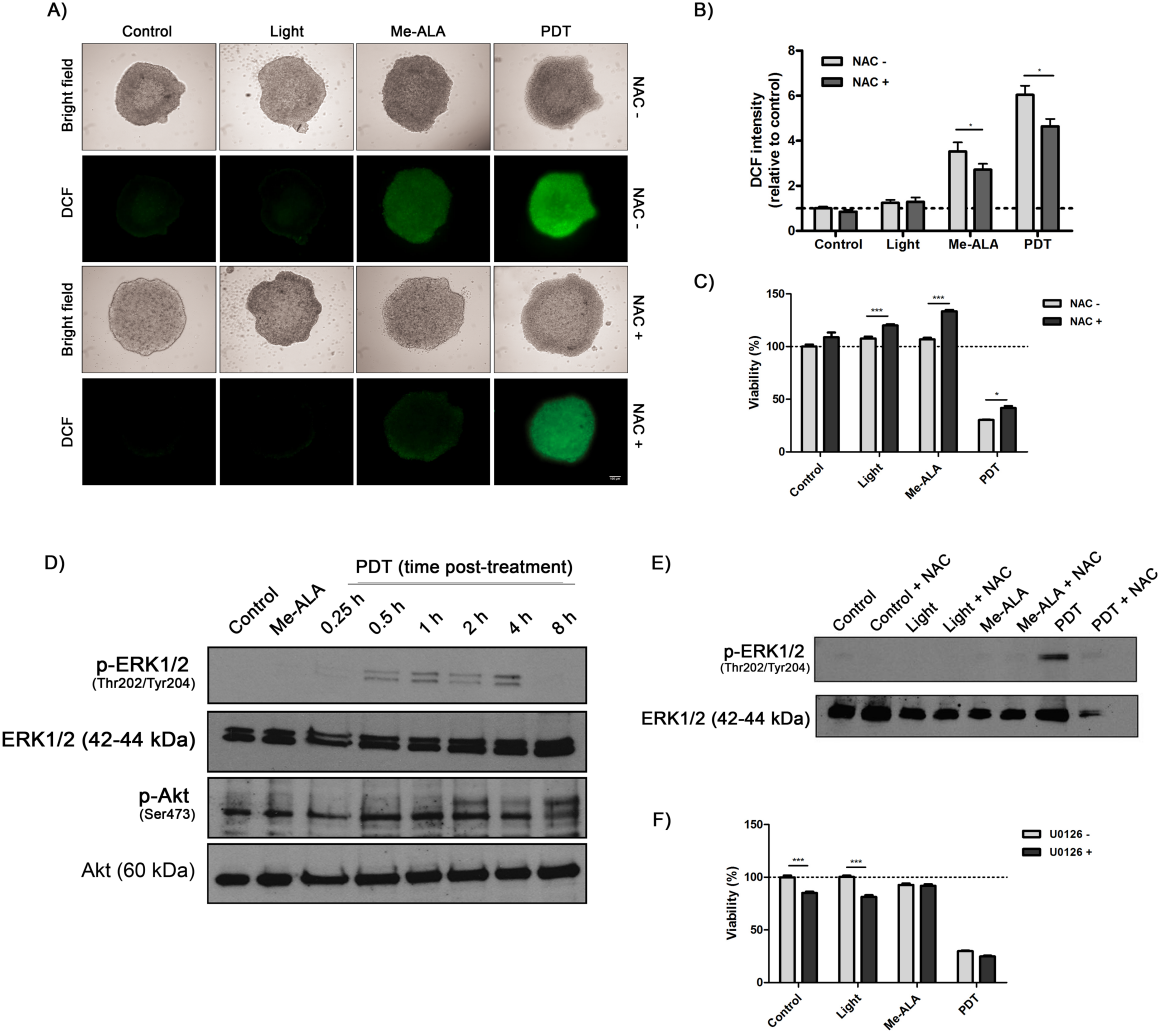
Oxidative stress modulates phosphorylation of ERK1/2 induced by photodynamic therapy. SW480 spheroids were incubated with Me-ALA (0.3 mM) in the presence or absence of NAC (30 mM) or U0126 (30 mM) for 24 h and then exposed to irradiation (0.7 J/cm^2^). ROS production, ERK1/2 and Akt phosphorylation and cell viability were analyzed 30 min, 0–8 h and 24 h post treatment, respectively. (A) Representative fluorescence microscopy images of DCF fluorescence. (B) DCF fluorescence intensity was measured using ImageJ software and referred to non-treated conditions. (C) Viability was evaluated by MTT assay 24 h post-PDT and referred to non-treated conditions. (D) Western blot was performed to detect phosphor-ERK1/2 and phosphor-Akt protein. The same membrane was stripped and reblotted for total ERK1/2 and total Akt as loading control. (E) Western blot was performed to detect phosphor-ERK1/2 1 hr post treatment. The same membrane was stripped and reblotted for total ERK1/2 as loading control. (F) Viability was evaluated by MTT assay 24 h post-PDT and referred to non-treated conditions. Control: Non-PDT treated cells; Light: Only irradiated cells; Me-ALA: Only Me-ALA-incubated cells; PDT: Photodynamic therapy-treated cells.

PDT takes advantage of the generation of ROS to generate focal tissue damage [1]. Concomitantly, it has been suggested that Akt and ERK activation may be a general signaling defense against oxidative stress and activation of these transduction pathway can confer a survival advantage to cells [25,26]. To explore the underlying mechanism on PDT resistance we measured ROS production using a fluorescent probe 2′,7′-dichlorofluorescin diacetate (DCF-DA), which reacts mainly with ROS to form a fluorescent compound at the point of interaction [20]. DCF staining was already evident 30 min after photodynamic treatment. Me-ALA-free spheroids exhibited basal ROS gradients from the periphery towards the center (Fig 2A). Generation of PpIX from the prodrug Me-ALA, despite not affecting cell viability, promoted the production of ROS in tumor spheroids (Fig 2A), which could be associated with previous kinetic studies revealing that PpIX itself induced a loss of mitochondrial transmembrane potential followed by ROS production [27]. On the other hand, PDT-treated colorectal spheroids showed an enhanced cellular oxidative stress (Fig 2A); both PDT and Me-ALA-mediated ROS generation was partially inhibited by the antioxidant *N*-acetylcysteine (NAC) (Fig 2A and 2B). In addition, the antioxidant was able to promote cell viability on irradiated, Me-ALA-incubated and PDT-treated colorectal *in vitro* microtumors (Fig 2C).

In order to clarify whether PDT activates ROS-related survival pathways, phosphorylation of ERK1/2 and Akt were examined in a time-dependent manner. The photodynamic effect on the phosphorylation of ERK1/2 is evident from 0.5 h to at least 4 h post-treatment, and decreased thereafter. There was not observed a significant activation of Akt post-treatment (Fig 2D and S1 Fig). ERK1/2 transient activation was observed only on PDT-treated spheroids (Fig 2E). Moreover, pretreatment with the antioxidant NAC markedly reduced PDT-induced ERK1/2 phosphorylation (Fig 2E). Surprisingly, photosensitivity was not affected when colorectal tumor spheroids were pretreated with the MEK1/2 inhibitor U0126 (Fig 2F). Inhibition of MEK by U0126 is selective as U0126 shows little, if any, effect on the kinase activities of other proteins. Further, U0126 has an approximately 100-fold-higher affinity for active MEK than do other previously identified MEK inhibitors [28]. These results clearly indicate that the high-level and lethal production of ROS by PDT is a potent and specific inducer of ERK1/2 pathway.

### 3.3. HIF-1α mediates the resistance of photodynamic treatment

**Fig 3:**
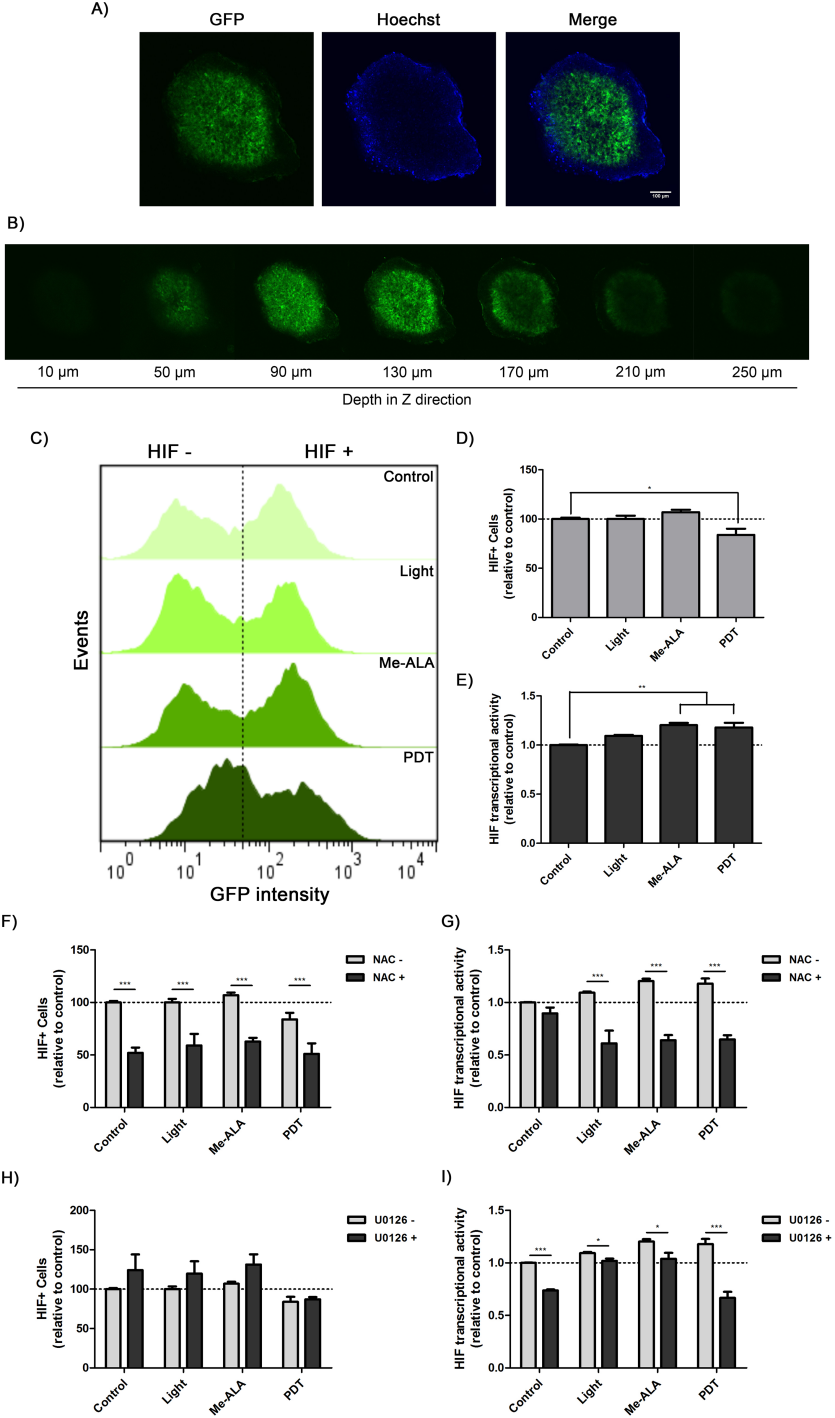
Oxidative stress-mediated induction of ERK1/2 phosphorylation increments HIF-1 transcriptional activity post-PDT on hypoxic colorectal tumor spheroids. SW480-HRE spheroids were incubated with Me-ALA (0.3 mM) in the presence or absence of U0126 (30 mM) or NAC (30 mM) for 24 h and then exposed to irradiation (0.7 J/cm^2^). After 12 h, 3D cultures were harvested and disaggregated, and GFP gene reporter fluorescence was analyzed. (A) Representative confocal microscopy images of the central layer of SW480-HRE spheroids before PDT. GFP + areas represented where HIF is active. For nuclear staining, spheroids were incubated with Hoechst dye for 1 h (blue). Bar = 100 μm. (B) Sequence of optical sections taken every 40μm of the spheroid before PDT.GFP + areas correlated with HIF activity. (C) Representative flow cytometry histograms of GFP expression. (D-F-H) HIF + cells were represented by GFP + cells, and referred to non-treated conditions. (E-G-I) HIF transcriptional activity of HIF + cells was indicated by GFP intensity, and referred to non-treated conditions. Control: Non-PDT treated cells; Light: Only irradiated cells; Me-ALA: Only Me-ALA-incubated cells; PDT: Photodynamic therapy-treated cells.

Given the increase in reactive oxygen species (ROS) and MAPKs signaling we inquiry if HIF-1 signaling, a known cascade regulated by ROS and MAPKs [29–31], was activated by PDT. We use SW480-HRE cells were used to generate spheroids. SW480-HRE stably expresses GFP under the control of an HRE element containing the DNA binding motif for HIF-1. Thus, GFP positive regions represented layers wherein HIF-1 was transcriptionally activated. It was demonstrated that SW480-HRE spheroids had a basal HIF + cells and showed a radial increase in HIF-1 activity from the periphery towards the center (Fig 3A and 3B). Accordingly with previous reported association between hypoxia and oxidative stress [32,33], the distribution of hypoxic areas within tumor spheroids was correlated with the pattern of ROS generation (Fig 2A). As oxygen distribution is altered by photodynamic consumption [1], it was proposed to evaluate whether lethal PDT regulates HIF-1 activity on colorectal spheroids. SW480-HRE represented a tool that allows monitoring the intensity of HIF-1 activity, following GFP expression by flow cytometry. For PDT-treated spheroids, the total HIF-1-expressing viable cells significantly decreased 12 h post-treatment (Fig 3C and 3D). On the opposite, quantification of HIF-1 reporter fluorescence intensity demonstrated that HIF-1 transcriptional activity on this population was enhanced by Me-ALA incubation or PDT treatment (Fig 3C and 3E). The inhibition of ROS production by NAC decreased the number of HIF-1 positive cells (HIF+) (Fig 3F) and HIF transcriptional activity of this population (Fig 3G), regardless treatment condition, suggesting that both basal and treatment-induced HIF modulation is regulated by ROS in 3D spheroids. In addition, U0126-mediated suppression of ERK1/2 activation diminished HIF-1 transcriptional activity of HIF+ cells (Fig 3H), although it did not affect the number of HIF-1+ cells (Fig 3I). Given that ERK inhibition had no significant effect on HIF-1 + cells, more PDT-resistant, it is consistent with the previous results showing that U0126 had no effect on photodynamic cytotoxicity (Fig 2F). Taken together, these results suggest that PDT induces HIF-1 transcriptional activation in SW480 spheroids through the pre-existing axis ROS → ERK1/2.

**Fig 4:**
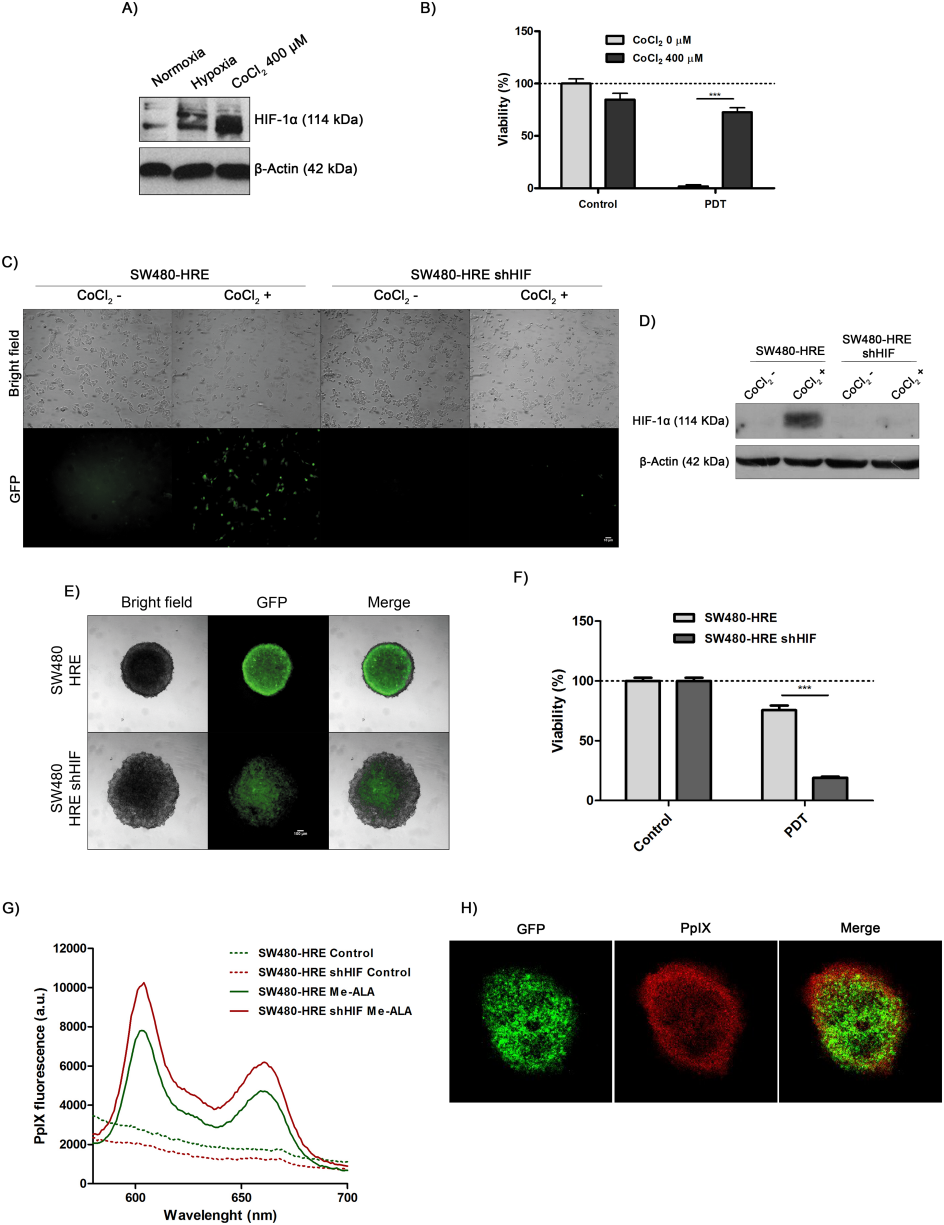
Upregulation of HIF-1α is associated with resistance to photodynamic treatment. (A) SW480 cells were incubated under normoxia (21% O_2_), hypoxia (cells were placed in a hypoxia chamber at 37°C, 5% CO_2_, and 1% O_2_) or with CoCl_2_ (Sigma, 400 μM) for 24 h. After that, western blot were performed to detect HIF-1α protein. The same membrane was stripped and reblotted for β-actin as loading control. (B) SW480 cells were pre-incubated with CoCl_2_ (400 μM) for 24 h. After that, they were treated with Me-ALA (0.3 mM) for 4 h and then exposed to lethal irradiation (0.3 J/cm^2^). Viability was evaluated by MTT assay 24 h post-PDT and referred to non-treated conditions (dotted line: 100% viability). SW480-HRE and SW480-HRE shHIF cells were incubated with CoCl_2_ (400 μM) for 24 h. (C) Representative fluorescence and light microscopy images. GFP + cells represents with HIF activity. Bar = 10 μm. D) Western blot was performed to detect HIF-1α protein. The same membrane was stripped and reblotted for β-actin as loading control. (E) Representative fluorescence and light microscopy images of spheroids generated from SW480-HRE and SW480-HRE shHIF cells. GFP + areas correlated with HIF activity. Bar = 100 μm. (F) SW480- HRE and SW480-HRE shHIF spheroids were incubated with Me-ALA (0.3 mM) for 24 h and then exposed to irradiation (0.2 J/cm^2^). Viability was evaluated by MTT assay 24 h post-PDT and referred to non-treated conditions. (G) SW480-HRE and SW480-HRE shHIF spheroids were incubated with Me-ALA. At 24 h, the intracellular PpIX was determined spectrofluorimetrically and normalized to cellular concentration. (H) Representative confocal microscopy images of the central layer of SW480-HRE spheroids after 24 h-Me-ALA incubation. GFP + areas correlated with HIF activity; PpIX shows red fluorescence. Control: Non-PDT treated cells; Me-ALA: Only Me-ALA-incubated cells; PDT: Photodynamic therapy-treated cells.

To define whether HIF-1 activation may be related to PDT cytotoxicity on SW480 cells, monolayers were incubated with CoCl_2_ and then subjected to lethal conditions of photodynamic treatment. As expected, the regulatory subunit HIF-1α was upregulated by hypoxia and by CoCl_2_, a well-known hypoxia-mimetic agent [34] (Fig 4A). CoCl_2_-mediated stabilization of HIF-1α was able to significantly confer resistance to PDT (Fig 4B). To further define the role of HIF-1α in making SW480 cells resistant to photodynamic treatment, RNA interference forHIF-1α was employed by stable transfection of cells with the specific siRNA against human HIF-1α mRNA. CoCl_2_ treatment failed to induce HIF-1 activity (Fig 4C) and to stabilize HIF-1α protein in SW480-HRE shHIF-1α clone (Fig 4D). HIF-1α silencing was associated with an increase in the diameter and partial loss of integrity of 3D structures. It was also observed a remarkable suppression of HIF-1 activity within SW480-HRE shHIF-1α spheroids, evidenced by a decrease in GFP reporter gene fluorescence (Fig 4E). Furthermore, inhibition of HIF-1α significantly restored the photosensitivity of hypoxic spheroids (Fig 4F), affecting the entire tridimensional structure from periphery to core center. More significantly, spectrofluorimetric analysis showed that intracellular PpIX content was enhanced by silencing HIF-1α (Fig 4G). This finding was further confirmed by confocal microscope images of Me-ALA-incubated SW480-HRE spheroids, which demonstrated that PpIX distribution inversely correlated with HIF-1 + areas (Fig 4G). These results indicate that HIF-1 induction negatively modulate the cytotoxicity of PDT on hypoxic areas of colorectal cancer (~900 μm from the oxygen source), which can be attributed at least in part to diminished photosensitizer accumulation.

## Discussion

Reactive oxygen species (ROS) are continuously generated within mammalian cells, as a direct consequence of the use of oxygen in aerobic respiration. The harmful effects of ROS are balanced by the action of endogenous antioxidant defense mechanisms. If the generation of free radicals exceeds the protective effects of antioxidants, indiscriminate oxidation causes damaging effects within cells, resulting in "oxidative stress" [35].

Oxidative stress-mediated damage has been used for developing antitumor therapies that provide an increase of lethal ROS in tumor cells. In this sense, photodynamic therapy (PDT) is a cancer therapy inducing tumor destruction via photosensitizer-mediated oxidative cytotoxicity. In PDT, *in-situ* photosensitization of a non-toxic sensitizer generates cytotoxic ROS that cause cell death of tumor, with minimal damage to the surrounding tissue. In general, treatment selectivity of PDT results from spatial confinement of light, drug and tissue oxygen within the irradiated region, potentially reducing the dose-limiting side effects [1]. PDT is becoming an ever more useful tool in oncology but is frequently restricted by the development of tumor hypoxic areas, limiting oxygen availability needed for photodynamic action [36]. Additionally, elevated ROS have been implicated in signal transduction pathways activation, associated with cellular survival, growth and proliferation [25].

Tumor hypoxia results from an inadequate oxygen supply by distant and abnormal cancer-associated vasculature and its consumption [37]. Hypoxia-inducible factor 1 (HIF-1) is a well-characterized transcription factor that regulates genes that are involved in crucial hallmarks of tumor biology, including cell survival, angiogenesis, anaerobic metabolism and invasion. HIF-1 consists of a constitutively expressed subunit HIF-1β and an oxygen-regulated subunit HIF-1α. The increased stability of HIF-1α under low O_2_ pressure is mainly regulated by post-translational modifications. Under normoxia, oxygen-dependent hydroxylation of HIF-1α subunit by prolyl hydroxylases (PHDs) signals their polyubiquitination and proteasomal degradation. In hypoxia, when less oxygen is available for PHDs-mediated hydroxylation, HIF-1α protein accumulates and translocates into the nucleus. There, it associates with HIF-1β and the co-activators p300/CBP to induce gene expression by binding to the conserved hypoxia-responsive element (HRE) [38,39]. In addition, the synthesis and activity of HIF-1α is regulated by mechanisms involving activation of the phosphoinositide 3-kinase (PI3K) and mitogen-activated protein kinase (MAPK) pathways. However, protein stabilization through hydroxylation inhibition is the major regulatory mechanism for HIF-1α induction [40].

To achieve functional and morphological equivalence to tumor hypoxic architecture, sophisticated 3D spheroids were developed, in order to recapitulate portions of the *in vivo* environment. Spheroids are a frequently used *in vitro* model of avascular tumor growth and the microenvironmental and physiological perturbations that occur in tumors [41]. We confirmed that 3D cell organization of SW480 colorectal cancer cells displayed a gradient of HIF activity from the center to the periphery (Fig 3A and 3B).

Considering the reduced effectiveness of photodynamic agents at lower O_2_ partial pressures and the rapid induction of tumor hypoxia by PDT itself by oxygen consumption [1], it may mean that this therapy is self-limiting. These discrepancies lead to the evaluation here of the role of PDT on different signaling pathways. Despite the fact that several survival pathways are activated in tumor cells post-PDT [42], we decided to explore those related to HIF-1 modulation [43], such as PI3K-Akt and MEK1/2-ERK1/2. Interestingly, despite ERK1/2 phosphorylation was absent in non-treated cells, activation of this pathway was clearly evident after PDT (0.5 to 4 h post-treatment) (Fig 2). Furthermore, it was successfully identified a pathway which appears to play a dominant role in regulating the photosensitized HIF-1 modulation on colorectal *in vitro* microtumors. It was revealed that oxidative stress generation after PDT was capable to upregulate HIF expression and HIF transcriptional activity (Fig 3F and 3G). Despite not affecting cell viability, production of ROS in Me-ALA incubated spheroids promoted HIF activity (Fig 3E), which was partially inhibited by NAC (Fig 3G). However, even if the axis ROS-HIF is active in Me-ALA incubated spheroids, this is not a reliable therapeutic situation: the availability of photosensitizer, light and oxygen and their relationship in time and space determines the efficacy of PDT [1]. Moreover, ERK1/2 phosphorylation positively modulated HIF-1 transcriptional activity after photodynamic treatment, without affecting the level of HIF-1 expressing cells (Fig 3H and 3I). A dual mechanism was proposed to explain how ROS could modulate HIF-1 activity. First, several reports confirmed that ROS stabilizes HIF-1α by inhibiting PHDs activity [44,45], since both superoxide anion and H_2_O_2_ are able to oxidize iron, essential cofactor of these enzymes [30,46,47]. Second, recent findings reveal that mitochondria-derived ROS are both required and sufficient to initiate HIF-1α stabilization during hypoxia, given that antioxidants maintained hydroxylation and prevented stabilization of alpha subunit during hypoxia [30,46,47]. On the other hand, some data demonstrated that HIF-1 phosphorylation by ERK1/2 promoted its nuclear accumulation and transcriptional activity by blocking its CRM1-dependent nuclear export [48]. Another study showed that ERK1/2 phosphorylation of p300/CBP coactivator enhanced its interaction with HIF-1, thereby incrementing HIF-1 transcriptional activity [49].

Hypoxia and overexpression of HIF-1 have been associated with radiation therapy and chemotherapy resistance, an increased risk of invasion and metastasis, and a poor clinical prognosis of solid tumors [50], including CRC [51]. In this study, we demonstrated that stabilization of HIF-1 by CoCl_2_ treatment or 3D-growing conditions protected cells to photodynamic damage (Figs 1A and 4B), whereas inhibition of this transcription factor promoted photosensitivity of tumor spheroids (Fig 4F). These findings, along with previous reports [52,53], illustrate the high concordance between HIF-1 and resistance to photodynamic treatment of solid tumors.

Photodynamic therapy involving prodrugs, such as aminolevulinic acid or its derivatives, may be further limited because conversion of the prodrug to the active photosensitizer appears to be less effective under hypoxic conditions [52]. In this work, the spatial pattern of Me-ALA-PDT treatment response (Fig 1D) correlated with PpIX accumulation, inversely associated with HIF-1 activity (Fig 4H). In addition, HIF-1α inhibition enhanced PpIX intracellular content (Fig 4G). Likewise, some data support the hypothesis that some enzymes involved in PpIX-heme biosynthetic pathway and PpIX accumulation are codified by HIF-regulated genes, such as ABCG2 [54–57], ferrochelatase [58,59], transferrin receptor [60,61] and heme-oxygenase [62,63].

In conclusion, this study adds another layer to the complexity of tumor hypoxic resistance to photodynamic treatment. Our results take a significant first step to the identification of the axis PDT → ROS → ERK1/2 on HIF-1 transcriptional activation and the role of HIF-1 pathways on photodynamic efficiency. These findings contributed to the discovery of key modulators of PDT resistance which can be applied to the design of combined therapeutics to target HIF directly or indirectly in order to optimize PDT-outcome on solid tumors.

## Supporting information

S1 Fig Effect of PDT on ERK1/2 and Akt phosphorylation. Densitometric analysis performed with the ImageJ software represented the signal intensity of phospho-ERK1/2 (A) and phospho-Akt (B) protein; the signal was normalized to total ERK1/2 and total Akt, respectively.
(TIF)

## Abbreviations

3D: Three dimensional
5-ALA: 5-aminolevulinic acid
CRC: Colorectal cancer
DCF: 2´,7´-dichlorofluorescein
DMSO: Dimethyl sulfoxide
FBS: Fetal bovine serum
GFP: Green fluorescent protein
H_2_DCFDA: 2´7´-dichlorodihydrofluorescein diacetate
HIF-1: Hypoxia inducible factor-1
HRE: Hypoxia-responsive element
MAPK: Mitogen-activated protein kinase
Me-ALA: Methyl-ALA
MTT: 1-(4,5-dimethylthiazol-2-yl)-3,5-diphenylformazan
NAC: *N*-acetylcysteine
PBS: Phosphate buffer saline
PDT: Photodynamic therapy
PHDs: Prolyl hydroxylases
PI3K: Phosphoinositide 3-kinase
PpIX: Protoporphyrin IX
PS: Photosensitizer
ROS: Reactive oxygen species
shHIF: Short hairpin RNA for HIF expression
siRNA: small interfering RNA
VEGF: Vascular endothelial growth factor
