# Supplementary figures and images for: Transcriptional activation of HIF-1 by a ROS-ERK axis underlies the resistance to photodynamic therapy
Source: PLoS One. 2017 May 17;12(5):e0177801. doi: 10.1371/journal.pone.0177801 (PMC5435305; doi:10.1371/journal.pone.0177801)

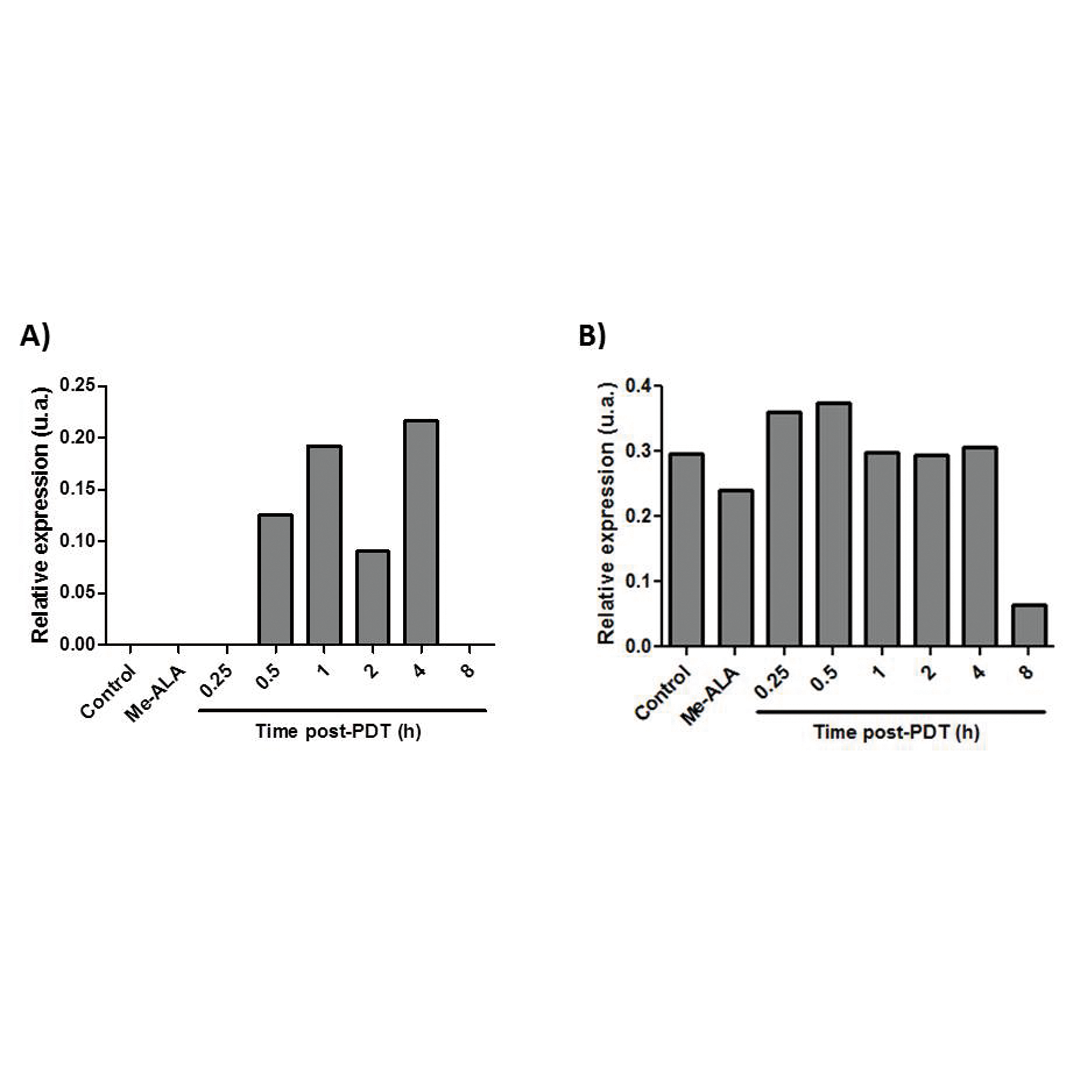

Supplement: S1 Fig — Densitometric analysis performed with the ImageJ software represented the signal intensity of phospho-ERK1/2 (A) and phospho-Akt (B) protein; the signal was normalized to total ERK1/2 and total Akt, respectively. (TIF) [file pone.0177801.s001.tif]
